# Approximating edit distances between complex tandem repeats efficiently

**DOI:** 10.1093/bioinformatics/btaf155

**Published:** 2025-04-09

**Authors:** Riki Kawahara, Shinichi Morishita

**Affiliations:** Department of Computational Biology and Medical Sciences, Graduate School of Frontier Sciences, The University of Tokyo, Chiba 277-8562, Japan; Department of Computational Biology and Medical Sciences, Graduate School of Frontier Sciences, The University of Tokyo, Chiba 277-8562, Japan

## Abstract

**Motivation:**

Extended tandem repeats (TRs) have been associated with 60 or more diseases over the past 30 years. Although most TRs have single repeat units (or motifs), complex TRs with different units have recently been correlated with some brain disorders. Of note, a population-scale analysis shows that complex TRs at one locus can be divergent, and different units are often expanded between individuals. To understand the evolution of high TR diversity, it is informative to visualize a phylogenetic tree. To do this, we need to measure the edit distance between pairs of complex TRs by considering duplication and contraction of units created by replication slippage. However, traditional rigorous algorithms for this purpose are computationally expensive.

**Results:**

We here propose an efficient heuristic algorithm to estimate the edit distance with duplication and contraction of units (EDDC, for short). We select a set of frequent units that occur in given complex TRs, encode each unit as a single symbol, compress a TR into an optimal series of unit symbols that partially matches the original TR with the minimum Levenshtein distance, and estimate the EDDC between a pair of complex TRs from their compressed forms. Using substantial synthetic benchmark datasets, we demonstrate that the estimated EDDC is highly correlated with the accurate EDDC, with a Pearson correlation coefficient of >0.983, while the heuristic algorithm achieves orders of magnitude performance speedup.

**Availability and implementation:**

The software program hEDDC that implements the proposed algorithm is available at https://github.com/Ricky-pon/hEDDC (DOI: 10.5281/zenodo.14732958)

## 1 Introduction

A class of genomic strings that consist of a series of duplicate string units (or motifs) are called tandem repeats (TRs, for short) (Smith 1976). TRs of 2–6 base units were reported in the early 1980s and were named microsatellites or short tandem repeats (Miesfeld and Krystal 1981, Spritz 1981, Hamada and Kakunaga 1982). Another class of TRs named minisatellites, duplicate units of a few dozen base pairs, were also found in 1985 (Jeffreys *et al.* 1985). In human populations, some micro/mini-satellites vary in length (Tautz *et al.* 1986) and have been valuable for understanding genetic diversity (Weber and Wong 1993, Bowcock *et al.* 1994) and for providing insight into disease-causing variants.

Indeed, over the past 30 years since 1990, approximately sixty diseases have been shown to be associated with extremely expanded TRs at different loci (Depienne and Mandel 2021, Rajan-Babu *et al.* 2024). Several disease-associated minisatellites have been identified (De Roeck *et al.* 2018, Song *et al.* 2018, Cortese *et al.* 2019, Course *et al.* 2020). Some disease-associated TRs have a complex structure in which different units are expanded in individual genomes (Koob *et al.* 1999, Liquori *et al.* 2001, Ishiura *et al.* 2018, Cortese *et al.* 2019), and are therefore difficult to characterize. Widely used tools such as TRF (Benson 1999) and RepeatMasker (http://repeatmasker.org) were not designed to handle this issue (Masutani *et al.* 2023).

With the availability of short-read sequencing data, a number of algorithms have been proposed to estimate the length and structure of TRs on a genome-wide basis in individual genomes (Dashnow *et al.* 2018, Dolzhenko *et al.* 2019, Mousavi *et al.* 2019). For example, 2529 distinct regions with complex TRs were identified by an analysis of 17 231 genomes of individuals with autism spectrum disorder and population controls (Trost *et al.* 2020). Although it is hard to accurately determine the entire structure of TRs of >100 bp in length using short-read sequencing, to solve this problem, algorithms for detecting complex TRs from long-read sequencing data have recently been proposed (Masutani *et al.* 2023).

**Figure 1.**
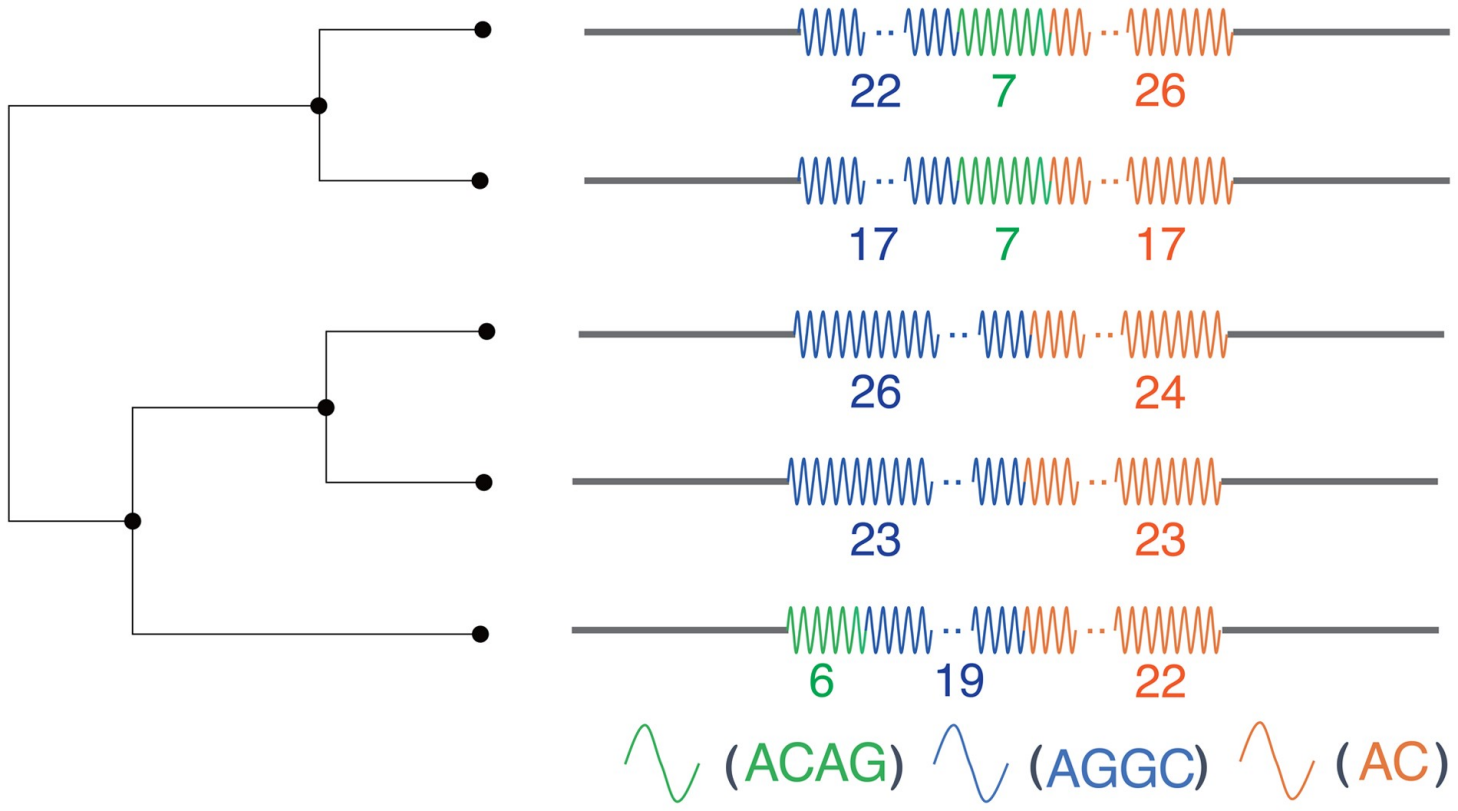
Schematic phylogenetic tree showing the evolution of five complex TRs with three different units, ACAG, AGGC, and AC, that are illustrated by green, blue, and orange waves. The number below each series of waves represents the number of unit occurrences; e.g. the bottom complex TR means ${\left(\mathrm{ACAG}\right)}_{6}{\left(\mathrm{AGGC}\right)}_{19}{\left(\mathrm{AC}\right)}_{22}$. These five complex TRs are located in an intron of *CNBP* at chr3:129 172 576–129 172 656 in the human reference genome(hg38) and are found in individual samples.

A population-scale analysis of long-read sequencing data recently has shown the evolution of high complex TR diversity (Ichikawa *et al.* 2023). This study shows that visualization of the phylogenetic tree of complex TRs is beneficial for understanding the diversity of complex TRs. (see Fig. 1). To do this, we need to measure the edit distance between pairs of complex TRs by considering duplication and contraction of units created by replication slippage. However, existing rigorous algorithms for this purpose are computationally expensive (Pinhas *et al.* 2013). Here, we propose a heuristic algorithm that approximates distances with high accuracy while achieving orders of magnitude performance speed-up.

## 2 Materials and methods

### 2.1 Complex tandem repeats

We define several technical terms and symbols to define complex tandem repeats (TRs). We use Σ to express a set of symbols, such as four nucleotides A,C,G and T. A string is a sequence of consecutive symbols in Σ, say AAAG. A set of strings over Σ is denoted by *U*; e.g. {AAAG,AG}.

A *tandem repeat* of string (unit or motif) *u* is defined as having the form $s{\left(u\right)}_{k}p$ such that *p* and *s* are a prefix and a suffix of *u*, respectively, and ${\left(u\right)}_{k}$ is a series of k(>1) copies of *u* or a single occurrence of *u* if *k *=* *1. For example, $\mathrm{AAG}{\left(\mathrm{AAAG}\right)}_{3}\mathrm{AA}$ is a tandem repeat of string (unit) AAAG. To eliminate ambiguity when expressing the same tandem repeat, assume that the unit of any tandem repeat is not a tandem repeat of any shorter substring. For example, AGAG cannot be a unit because it is a tandem repeat of unit AG. A tandem repeat of *u* is maximal if none of its superstrings are tandem repeats of *u*. In AA  AGAGAGAG  GAGG, the underlined substring is a “maximal” tandem repeat ${\left(\mathrm{AG}\right)}_{4}$.

A concatenation of one or more tandem repeats of units in *U* is called a *complex tandem repeat* of *U*; e.g. ${\left(\mathrm{AAAG}\right)}_{3}{\left(\mathrm{AG}\right)}_{2}{\left(\mathrm{AGGG}\right)}_{3}{\left(\mathrm{AG}\right)}_{2}{\left(\mathrm{AAAG}\right)}_{3}$. The concatenation of two strings *u* and *v* can be expressed explicitly with u·v, but the concatenation symbol · can be omitted for readability if the string concatenation, say *uv*, can be parsed correctly.

We here present some tips to reduce ambiguity in expressing tandem repeats. A unit *v* is a *rotation* of *u* if *u* is a concatenation of nonempty prefix *p* and suffix *q* (i.e. *u* = *pq*), and *v* is the concatenation of *q* followed by *p* (namely, *v* = *qp*). Addition of all rotations to *U* increases the number of ways to represent a given string as complex TRs of *U*. For example, when S=AGAGAGAG and U={A,G,AG,GA}, we can generate various concatenations of *U* for *S*; e.g. ${\left(\mathrm{AG}\right)}_{4}$ and $A{\left(\mathrm{GA}\right)}_{3}G$. To reduce ambiguity in the expression, a smaller unit set should be used. For example, setting U={AG} yields $S={\left(\mathrm{AG}\right)}_{4}$ only. To this end, it is effective to use a *bifix-free* unit such that any prefix and suffix of length k(≥1) do not match for all *k* less than the length of the unit. For example, AAAG and GAAA are bifix-free but AAGA and AGAA are not. Experimental results also show that bifix-free units significantly reduce the unit set (Masutani *et al.* 2023).

### 2.2 Impure complex tandem repeats

If string *S* matches a complex TR, *S* is defined as *pure*; otherwise, *S* is *impure*. In reality, impure complex TRs are common because pure complex TRs can contain substitutions, insertions, and deletions during evolution. When *S* is impure and partially matches a complex TR of *U*, their difference is of interest. A common approach to measure the difference is the Levenshtein distance (i.e. the sum of substitutions, insertions, and deletions) (Levenshtein 1966) of the global alignment between *S* and the complex TR. When *S* can partially match more than one complex TR of *U*, it is ideal to select an optimal one that minimizes the Levenshtein distance. For example, consider

S=AAAG AAAG AAG AG AAAG AAAG

When U={AAAG,AG}, *S* partially matches ${\left(\mathrm{AAAG}\right)}_{2}{\left(\mathrm{AG}\right)}_{2}{\left(\mathrm{AAAG}\right)}_{2}$ with one deletion of A in AAG, and ${\left(\mathrm{AAAG}\right)}_{5}$ with one deletion of G in AAG. Thus, both of the two complex TRs are optimal solutions.

For calculating optimal solutions efficiently, we revisit the approximate regular expression matching problem to compute an optimal instance of a regular expression that maximizes its alignment score with a given string, because a concatenation of elements in *U* is represented as the regular expression $(u_{1}|\ldots |u_{k})*$ for all units $u_{1},\ldots ,u_{k}$ in *U*. The approximate regular expression matching problem can be solved efficiently in $O(|S|\cdot \sum_{u\in U}|u|)$ (Myers and Miller 1989). This concept has recently been also used in the string decomposer algorithm (Dvorkina *et al.* 2020), which is a wraparound dynamic programming algorithm that handles a set of multiple repeat units, say *U*.

### 2.3 Edit distance with duplication and contraction of units

Let us consider the problem of generating a phylogenetic tree of impure complex TRs $S_{1},\ldots ,S_{n}$. This construction requires knowing the distance between *S_i_* and *S_j_*, denoted by $d(S_{i},S_{j})$, for all pairs of strings. A simple distance, say the minimum Levensthtein distance between *S_i_* and *S_j_*, can be used for $d(S_{i},S_{j})$; however, since DNA slippage is as frequent as substitutions and indels in complex TRs, it is essential to consider two DNA slippage operations, duplications and contractions of units, in addition to substitutions, insertions, and deletions of symbols in Σ. To this end, we first consider the ideal situation that a TR is pure and perfectly matches a complex TR, before showing how to handle the impure case. For example, when U={AAAG,AG}, consider source string *S* and target string *T*:

$$S={\left(\mathrm{AAAG}\right)}_{3}{\left(\mathrm{AG}\right)}_{2}{\left(\mathrm{AAAG}\right)}_{5}\;\;\text{and}\;\;T={\left(\mathrm{AAAG}\right)}_{5}{\left(\mathrm{AG}\right)}_{5}{\left(\mathrm{AAAG}\right)}_{3}$$

*S* can be converted to *T* by duplicating the unit (AAAG) twice, duplicating the unit (AG) trice, and contracting the unit (AAAG) twice. These unit editing operations, duplication and contraction of units, help us understand how replication slippage change tandem repeats.

In real situations, strings can be impure and partially match complex TRs of *U*. In this setting, in addition to duplication and contraction of units, we need to use the three fundamental edit operations substitution, insertion, and deletion of single nucleotides (Bérard and Rivals 2003). When string *S* = *W*_0_ is transformed into string *T* = *W_n_* by a sequence of *n* edit operations $W_{0}\to W_{1}\to \ldots \to W_{n}$, let *c_i_* denote the nonnegative cost of the *i*th operation $W_{i-1}\to W_{i}(i=1,\ldots ,n)$, and call the sum, $\sum_{i=1}^{n}c_{i}$, the edit distance by the edit operation sequence.

For example, if the *i*th edit operation is a substitution, an insertion, or a deletion of a single letter, we can set *c_i_* = 1 following the idea of the Levenshtein distance. If the *i*th edit operation duplicates unit *α* in *W_i_* (or contracts αα in *W_i_* into *α*, respectively), the unit duplication (contraction) operation is equivalent to a series of |α| consecutive insertions (deletions). Assuming that the former is more likely to happen than the latter in tandem repeats, to favor the unit duplication (contraction), we can set *c_i_* to a value smaller than |α|; e.g. $c_{i}=|\alpha |/2$. Actually, we use this cost setting as the default in our program. The minimum distance between two strings using among all possible sequences of edit operations is called the *edit distance with duplication and contraction* (*EDDC*, for short).

Let *q* denote the length of the longest unit in *U*, and *n* denote the sum of the lengths of the two input strings. An $\left((|\Sigma |+|U|)(n^{3}+n^{2}q^{2})\right)$-time algorithm for computing the EDDC is proposed in Pinhas *et al.* (2013). In practice, as *n* and *q* grow to, say, thousands, the algorithm becomes computationally expensive and infeasible.

### 2.4 A heuristic, efficient algorithm for estimating EDDC

We here propose an efficient heuristic algorithm for estimating the EDDC between impure complex TRs. Our idea is to prioritize unit duplication and contraction over insertion and deletion explicitly. We convert each string *S* to a continuous sequence of units in *U*, denoted by $\bar{S}$, that matches *S* with the minimum Levenshtein distance by using the $O(|S|\cdot \sum_{u\in U}|u|)$-time string decomposer algorithm (Dvorkina *et al.* 2020). The units in $\bar{S}$ are treated as single symbols to compress *S* effectively when the units are long. In the running example, let α=AAAG and β=AG. The two strings on the left are compressed into the strings on the right:

$$\begin{array}{c} S={\left(\mathrm{AAAG}\right)}_{3}{\left(\mathrm{AG}\right)}_{2}{\left(\mathrm{AAAG}\right)}_{5}\to \bar{S}={\left(\alpha \right)}_{3}{\left(\beta \right)}_{2}{\left(\alpha \right)}_{5}\\ T={\left(\mathrm{AAAG}\right)}_{5}{\left(\mathrm{AG}\right)}_{5}{\left(\mathrm{AAAG}\right)}_{3}\to \bar{T}={\left(\alpha \right)}_{5}{\left(\beta \right)}_{5}{\left(\alpha \right)}_{3} \end{array}$$

This unit representation facilitates the design of a heuristic algorithm that emphasizes unit duplication and contraction over the other edit operations. To estimate the EDDC between strings *S* and *T*, we calculate a distance between optimal complex TRs, $\bar{S}$ and $\bar{T}$, that are defined above by limiting the application of the five edit operations “only” to units in *U* so as to reduce the actual calculation time.

An algorithm implementing this idea is detailed below. As $\bar{S}$ and $\bar{T}$ are series of symbols for units in *U*, we denote each unit explicitly and let $\bar{S}=u_{0}\ldots u_{n-1}$ and $\bar{T}=v_{0}\ldots v_{m-1}$. Let half-open interval u[i,j)(i≤j) represent the substring starting at position *i* and ending at position *j–*1. In particular, u[0,0) is the empty string denoted by *ϵ*. We design a method for calculating a distance between $\bar{S}=u[0,n)$ and $\bar{T}=v[0,m)$ by the following recursive function *dp*(*i*, *j*), which represents an alignment score between u[0,i) and v[0,j) using for nonnegative integers *i* and *j*:

$$dp(i,j)=\{\begin{array}{c} f(v,0,j,\epsilon )\;\;\;\;\text{if}\;i=0\\ f(u,0,i,\epsilon )\;\;\;\;\text{if}\;j=0\\ \underset{x\in U}{\min}\underset{0\leq p<i}{\min}\underset{0\leq q<j}{\min}\\ \;\;\;\;\;\;\{dp(p,q)+f(u,p,i,x)+f(v,q,j,x)\}\\ \;\;\;\;\;\;\;\;\;\;\;\;\;\;\;\text{otherwise} \end{array}$$

where f(w,b,e,z) is a recursive function that converts the interval of units w[b,e)  (b≤e) in string *w* to unit z∈U with the minimum sum of the “cost” values defined below:

$$f(w,b,e,z)=\{\begin{array}{c} 0\;\;\;\;\;\;\;\;\;\;\;\;\;\;\;\;\;\;\text{if}\;e=b\\ \text{cost}(w[b]\to z)\;\;\;\;\text{if}\;e=b+1\\ \underset{b<k\leq e}{\min}\underset{x\in U}{\min}\underset{y\in U}{\min}\\ \;\;\;\;\;\;\{f(w,b,k,x)+f(w,k,e,y)+\text{cost}(xy\to z)\}\\ \;\;\;\;\;\;\;\;\;\;\;\;\;\;\;\;\;\;\;\text{otherwise} \end{array}$$

**Figure 2.**
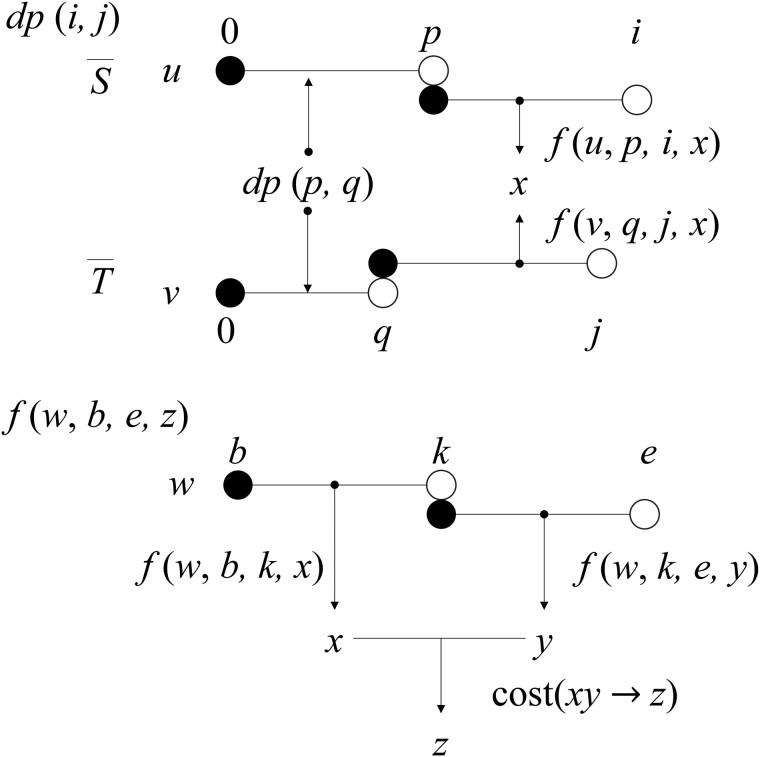
Schematic figure to illustrate how *dp* and *f* work. While *dp* calculates an alignment score between unit intervals, *f* partitions a unit interval into individual units recursively, and applies the five edit operations to units to compute the minimum sum of cost values.

Figure 2 illustrates how *dp* and *f* work. In the second formula of *f*, cost(w[b]→z) denotes the EDDC between w[b]∈U and z∈U, and hence, the costs of the five edit operations are considered here. In the last formula, w[b,k) and w[k,e) are transformed to x∈U and y∈U respectively at minimum cost, followed by *xy* to *z*. cost(xy→z) is the EDDC between *xy* and *z* for x,y,z∈U. A table of “cost,” which is actually a table of EDDC values, needs to be calculated in advance for units in *U*. If the number of units is small, the computation time for this table will also be short, and we will consider this issue in the next subsection.

To explain the time complexity analysis concisely, we assume that $\bar{S}=u_{0}\ldots u_{n-1}$ and $\bar{T}=v_{0}\ldots v_{m-1}$ are of the same length (*m *=* n*). The worst-case time complexity of computing *f* is $O(n^{3}|U{|}^{3})$ because there are $O(n^{2})$ pairs of *b* and *e* such that 0≤b<e≤n, O(|U|) instances of z∈U, *O*(*n*) instances of *k*, and $O(|U{|}^{2})$ pairs of units x,y∈U. Here we improve the performance of the algorithm. Since y∈U does not occur in f(w,b,k,x), by computing $\min_{y\in U}\{f(w,k,e,y)+\text{cost}(xy\to z)\}$ for all possible values of (k,e,x,z) in advance, we have:

$$\begin{array}{c} f(w,b,e,z)=\underset{b<k\leq e}{\min}\underset{x\in U}{\min}(f(w,b,k,x)\\ +\underset{y\in U}{\min}\{f(w,k,e,y)+\text{cost}(xy\to z)\}), \end{array}$$

which reduces the complexity to $O(n^{3}|U{|}^{2}+n^{2}|U{|}^{3})$ from $O(n^{3}|U{|}^{3})$. Indeed, the computational performance analysis in the next section shows a remarkable reduction. Similarly, in the last formula of the definition of *dp*(*i*, *j*), *q* does not appear in f(u,p,i,x). By computing $\min_{0\leq q<j}(dp(p,q)+f(v,q,j,x))$, which is denoted by g(v,p,j,x) below, beforehand, we have:

$$dp(i,j)=\underset{x\in U}{\min}\underset{0\leq p<i}{\min}(f(u,p,i,x)+g(v,p,j,x))$$

The time complexity of calculating g(v,p,j,x) is $O(n^{3}|U|)$ because the ranges of *p*, *q*, *j* and *x* are 0≤p<i, 0≤q<j, i<j≤n, and x∈U. The time complexity of computing *d*(*i*, *j*) is $O(n^{3}|U|)$ as well because variables are *i*, *j*, *p*, and *x*. Of note, *n* is the length of $\bar{S}$ and is typically smaller than the length of *S* because $\bar{S}$ is a compressed form of *S*. Thus, in practice, this heuristic algorithm runs faster than the algorithm for computing the accurate EDDC (Pinhas *et al.* 2013). Later we will show such examples in Fig. 5C.

### 2.5 Better estimation by adding unit variants in impure complex tandem repeats

To better estimate the EDDC between complex TRs, we note that impure complex TRs may actually have many mutations, thereby generating “variants” of units. Variants of some units may spread during evolution and can be commonly shared among individuals. Adding those unit variants to unit set *U* is expected to produce a better approximation of the EDDC between impure complex TRs. For example, given U={AAAG,AG} and

S=AAAG AAAG AAG AG AAAG AAAG,

${\left(\mathrm{AAAG}\right)}_{2}{\left(\mathrm{AG}\right)}_{2}{\left(\mathrm{AAAG}\right)}_{2}$ and ${\left(\mathrm{AAAG}\right)}_{5}$ are two optimal, impure complex TRs. Addition of AAG to *U* sets U={AAAG,AG,AAG}, and *S* perfectly matches ${\left(\mathrm{AAAG}\right)}_{2}\mathrm{AAG}\cdot \mathrm{AG}{\left(\mathrm{AAAG}\right)}_{2}$ and is pure. Removal of AG from *U* sets U={AAAG} and yields a single, optimal, impure complex TR ${\left(\mathrm{AAAG}\right)}_{5}$.

Care must be taken not to add too many unit variants in *U* to avoid blowing up computation time. Put another way, we are facing Occam’s razor problem; namely, a larger unit set can generate more accurate complex TRs with fewer discrepancies, but can increase the computation time, which is a trade-off between accuracy and computational time. In particular, this issue becomes crucial when cost(xy→z) is calculated for all possible instances of x,y,z∈U when *U* grows. To reduce the computational time, it is effective to eliminate rule xy→z such that *x* and *y* can be converted to *p* and *q* respectively, and the total cost of conversions (the right side of the following inequality) is smaller than the cost of the focal rule in the left side; namely,

cost(xy→z)>cost(x→p)+cost(y→q)+cost(pq→z).

In what follows, this heuristic algorithm for estimating the EDDC between impure complex TRs is called *hEDDC*, while the accurate algorithm for computing the EDDC (Pinhas *et al.* 2013) is called *EDDC_exact*.

## 3 Results

### 3.1 Empirical analysis of estimating EDDC

From a theoretical perspective, it is ideal to rigorously analyze the approximation rate of the estimated EDDC output by hEDDC to the accurate EDDC. We do not have clear answers to this question partly because the analysis depends on the selection of unit set *U*. In this study, we assume that *U* is given, but it is an open question whether computing the optimal unit set is computationally tractable or not (Masutani *et al.* 2023). We instead use a bench mark data to demonstrate how hEDDC outputs distances that are highly correlated with the distances computed by EDDC_exact. We use a bench mark dataset with five complex TR patterns:

-
${\left(\mathrm{AAAG}\right)}_{i}{\left(\mathrm{AG}\right)}_{j}$ ${\left(\mathrm{CAG}\right)}_{i}{\left(\mathrm{CAA}\right)}_{j}$  ${\left(\mathrm{AAAG}\right)}_{i}{\left(\mathrm{AG}\right)}_{j}{\left(\mathrm{AAAG}\right)}_{k}$
-
${\left(\mathrm{AAAG}\right)}_{i}{\left(\mathrm{AG}\right)}_{j}{\left(\mathrm{AAAG}\right)}_{k}{\left(\mathrm{AG}\right)}_{l}{\left(\mathrm{AAAG}\right)}_{m}$

-
${\left(\mathrm{AGGGG}\right)}_{i}{\left(\mathrm{AAAAGAAAGAGAGGG}\right)}_{j}{\left(\mathrm{AGGGG}\right)}_{k}$

Individual complex TR pattern has two or more units, and each unit in parentheses is associated with a variable (i,j,k,l and *m*) that shows the number of unit occurrences. To generate a variety of *m* instances of complex TR pattern, random values ranging from 0 to 50 are assigned to each variable independently. Next, we generate impure complex TRs from pure complex TRs to see how sequencing errors affect the prediction of the original pure complex TR patterns. To do this, we modified letters of strings at random positions by sequencing errors (substitutions, insertions, and deletions) at the rate of 0%, 1%, 3%, 5%, and 10%. For each of the 5 complex TR patterns, 5 datasets with different error rates are generated, so a total of 25 datasets are used to compare the distances that hEDDC and EDDC_exact output.

In each dataset with *m* complex TRs, we calculated the distances between m(m−1)/2 pairs of different repeats using hEDDC and EDDC_exact. We ignored a pair of identical repeats because each algorithm outputs the zero distance between the same repeats. Since each dataset has different complex TRs with different lengths, the distance between two complex TRs, *S* and *T*, largely depends on their lengths, |S| and |T|. To facilitate comparison, the distances are normalized by dividing them by $\sqrt{\left|S|\cdot |T\right|}$. The Pearson correlation coefficient of the normalized distances output by hEDDC and EDDC_exact is used to measure how they are consistent. Because EDDC_exact is very slow in calculating distances and can process 20 complex TRs in a reasonable time, datasets with 20 complex TRs are used.

**Figure 3.**
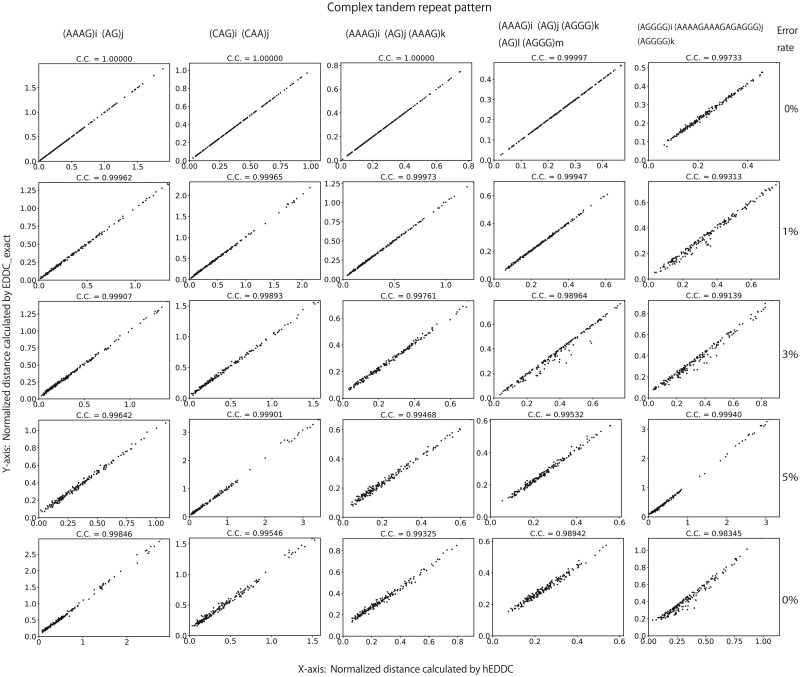
A dot in each plot represents a pair of the normalized distances calculated by hEDDC in the *x*-axis and EDDC_exact in the *y*-axis. The Pearson correlation coefficient (C.C., for short) of dots is shown at the top of each plot. The five columns show the five complex TR patterns, while the five rows show the error rate of 0%, 1%, 3%, 5%, and 10%. Input fasta files containing all TRs, programs to process the input, and output PDF files are available at https://github.com/Ricky-pon/hEDDC/.

Figure 3 shows dot plots of 25 datasets such that the columns represent the five complex TR patterns and the rows show the error rate of 0%, 1%, 3%, 5%, and 10%. In each plot, a dot shows a pair of the normalized distances calculated by hEDDC in the *x*-axis and EDDC_exact in the *y*-axis. The Pearson correlation coefficient is shown at the top of each plot. As the error rate increases, the Pearson correlation coefficient becomes lower but is sufficiently high and is >0.983 in all datasets. This demonstrates that hEDDC and EDDC_exact are consistent.

**Figure 4.**
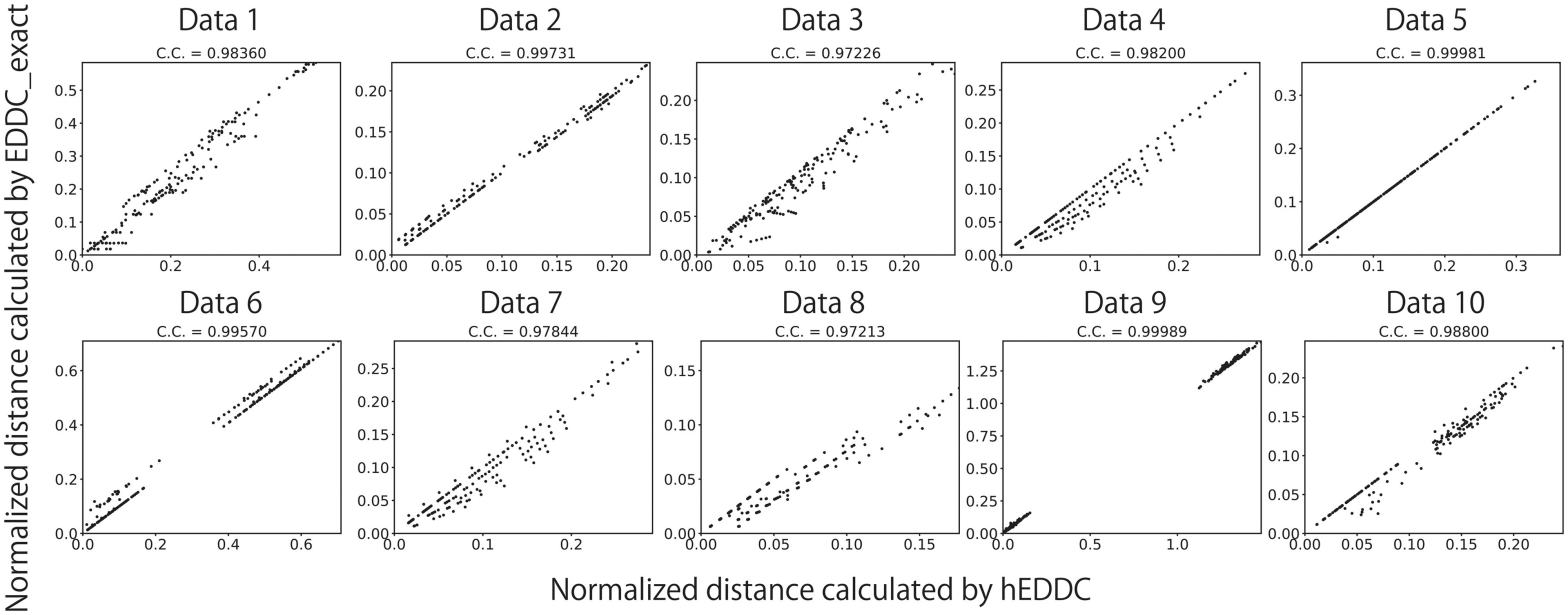
Similar to Fig. 3, plots are calculated for ten real datasets that are available at https://github.com/Ricky-pon/hEDDC/.

In Fig. 4, we also compared the two algorithms using approximately 200 tandem repeat pairs across 10 real datasets and found that the two algorithms produced highly correlated results from these real examples. Pearson correlation coefficient ranges from 0.972 to 0.999.

A phylogenetic tree can be generated from a normalized distance matrix, and the code for this purpose can be found at https://github.com/Ricky-pon/hEDDC/.

### 3.2 Computational performance

We will show hEDDC outperforms EDDC_exact by orders of magnitude in terms of user time using the Apple M1 Max processor (10 high-performance cores, clocked at 3.228 GHz) and 64 GB of main memory.

**Figure 5.**
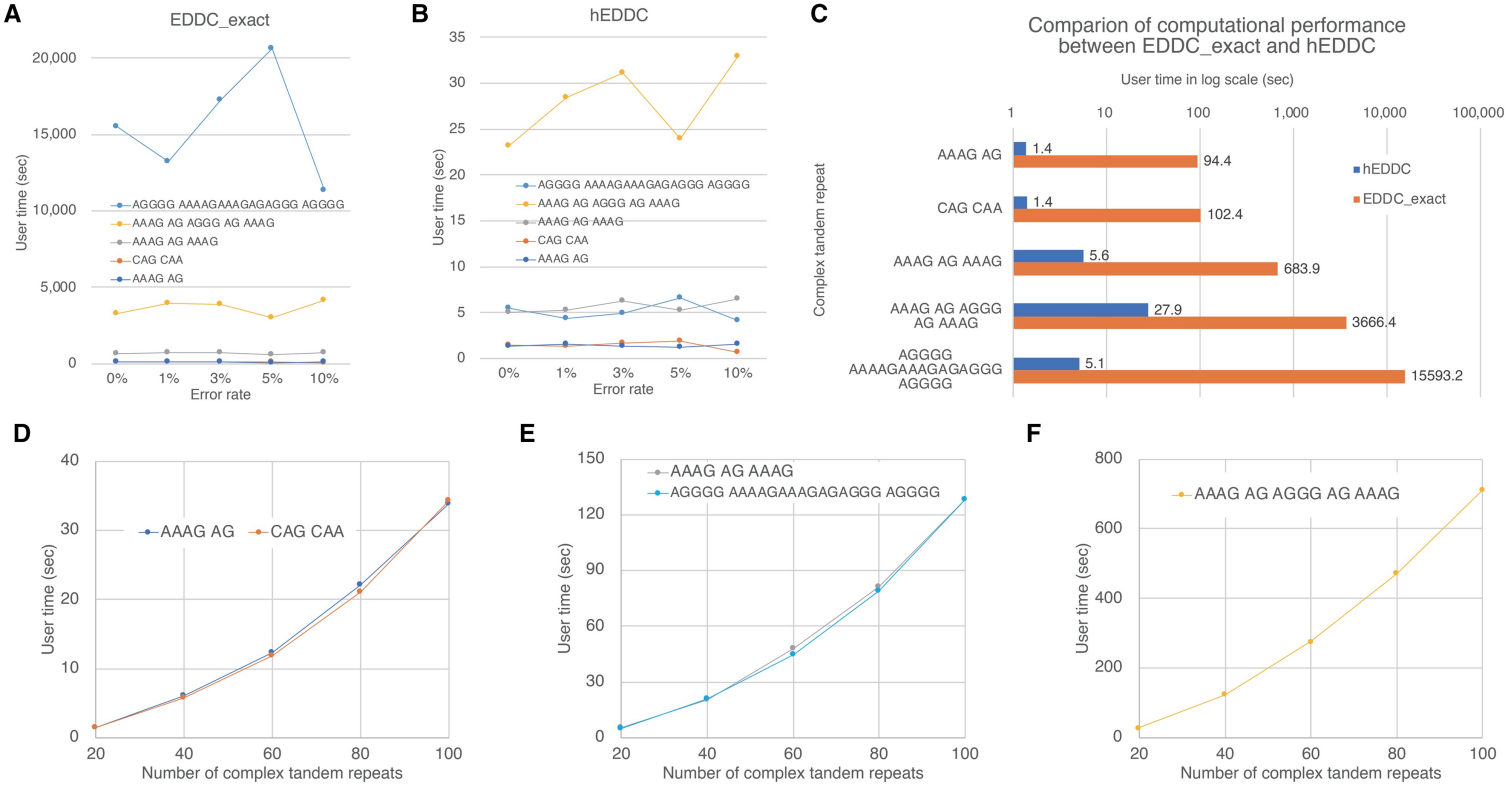
Computational performance: (A and B) User time of EDDC_exact (A) and hEDDC (B) applied to 25 datasets with 20 complex TRs. (C) Comparison of EDDC_exact and hEDDC in terms of the average user time grouped by the five complex TR patterns. (D–F) Computational performance (user time in the *y*-axis) of hEDDC to process five datasets with 20, 40, 60, 80, and 100 complex TRs in the *x*-axis.

For this comparison, datasets with 20 complex TRs were used because EDDC_exact was very slow to handle patterns of ${\left(\mathrm{AGGGG}\right)}_{i}{\left(\mathrm{AAAAGAAAGAGAGGG}\right)}_{j}{\left(\mathrm{AGGGG}\right)}_{k}$. Figure 5A and B respectively show user time of EDDC_exact and hEDDC for the 25 datasets. Because the user time was not correlated with the error rate in each complex TR pattern, we calculated the average user time of all error rates and compared the averages between EDDC_exact and hEDDC in Fig. 5C. hEDDC achieved orders of magnitude performance speedup while maintaining a high Pearson correlation coefficient. Figure 5C also shows that hEDDC is effective in reducing computation time during processing when it handles ${\left(\mathrm{AGGGG}\right)}_{i}{\left(\mathrm{AAAAGAAAGAGAGGG}\right)}_{j}{\left(\mathrm{AGGGG}\right)}_{k}$ because it can compress the three longer units into three single symbols.

Figures 5D–F show the computation performance of hEDDC to process datasets with *m* complex TRs (for m= 20, 40, 60, 80, and 100). The user time is approximately proportional to the square of *m* because m(m−1)/2 pairs of different repeats are considered. Figure 5E exhibits that two patterns containing three units have similar computational performance even if the units in one pattern are shorter than the units in the other, demonstrating that hEDDC successfully compresses longer units in this example. In contrast, Fig. 5F indicates that hEDDC gets slower when processing complex TRs with five units.

## 4 Conclusion and discussion

Because the EDDC between complex TRs is costly to compute (Pinhas *et al.* 2013), we have proposed a heuristic EDDC algorithm named hEDDC for estimating the EDDC. Experimental results using a variety of synthetic benchmark data show the estimated EDDC is correlated with the accurate EDDC (with a Pearson correlation coefficient of >0.983), while hEDDC is faster than EDDC_exact by orders of magnitude.

Computing optimal phylogenetic trees of complex tandem repeats from edit distances is another serious problem; however, there is no known efficient solution to this problem, so there is no definitive answer. More precisely, a phylogenetic tree is an unordered binary tree, the two children of any internal node are unordered, and computing the optimal unordered binary tree is MAX SNP hard in terms of tree edit distance (Bille 2005). Due to this intractability, measuring the exact similarity between two phylogenetic trees generated from exact and approximate edit distance matrices can be computationally expensive.

The user time of hEDDC scales as the square of the number of complex TRs (Fig. 5D–F) because hEDDC calculates the distances between all pairs of TRs to visualize a phylogenetic tree. Although hEDDC can handle hundreds of complex TRs, processing thousands of TRs requires a large amount of computational time. To reduce the problem size, e.g. complex TRs can be clustered into hundreds of TRs according to length and unit composition in advance. In hEDDC, the costs of the five edit operations (substitution, insertion, deletion, unit duplication, and unit contraction) are set to somewhat ideal values (see Section 2.3). However, to study the evolution of high complex TR diversity at individual loci precisely, a large number of complex TRs will need to be collected from populations in a genome-wide manner, and the costs should be carefully considered and optimized by taking account of sequence composition in units.

Indeed, population-scale analysis of short/long-read sequencing data has recently revealed a large number of hidden complex TRs in a genome-wide manner (Trost *et al.* 2020, Ichikawa *et al.* 2023). Increasing long-read sequencing data generation using PacBio and Nanopore sequencers may require processing millions of individuals to study the high diversity of complex TRs in the near future.
